# Galaxy Helm chart: a standardized method for deploying production Galaxy servers

**DOI:** 10.1093/bioinformatics/btae486

**Published:** 2024-08-06

**Authors:** Nuwan Goonasekera, Alexandru Mahmoud, Keith Suderman, Enis Afgan

**Affiliations:** Australian BioCommons, University of Melbourne, Melbourne, VIC 3052, Australia; Channing Division of Network Medicine, Harvard Medical School, Boston, MA 02115, United States; Department of Biology, Johns Hopkins University, Baltimore, MD 21210, United States; Department of Biology, Johns Hopkins University, Baltimore, MD 21210, United States

## Abstract

**Motivation:**

The Galaxy application is a popular open-source framework for data intensive sciences, counting thousands of monthly users across more than 100 public servers. To support a growing number of users and a greater variety of use cases, the complexity of a production-grade Galaxy installation has also grown, requiring more administration effort. There is a need for a rapid and reproducible Galaxy deployment method that can be maintained at high-availability with minimal maintenance.

**Results:**

We describe the Galaxy Helm chart that codifies all elements of a production-grade Galaxy installation into a single package. Deployable on Kubernetes clusters, the chart encapsulates supporting software services and implements the best-practices model for running Galaxy. It is also the most rapid method available for deploying a scalable, production-grade Galaxy instance on one’s own infrastructure. The chart is highly configurable, allowing systems administrators to swap dependent services if desired. Notable uses of the chart include on-demand, fully-automated deployments on AnVIL, providing training infrastructure for the Bioconductor project, and as the AWS-recommended solution for running Galaxy on the Amazon cloud.

**Availability and implementation:**

The source code for Galaxy Helm is available at https://github.com/galaxyproject/galaxy-helm, the corresponding Helm package at https://github.com/CloudVE/helm-charts, and the required Galaxy container image https://github.com/galaxyproject/galaxy-docker-k8s.

## 1 Introduction

The Galaxy application allows anyone to aggregate data from various sources, run thousands of specialized tools, build multi-step workflows, and access a variety of compute resources—all through a user-friendly graphical web interface (Galaxy Community 2024). Behind this accessible interface lies a complex ecosystem of software that includes Galaxy, a database, a job manager, a file server and reference data, a message queue, a metrics server, thousands of tools, and a host of interconnecting configurations. In addition, Galaxy deployments also need user authentication and access control, active monitoring, and regular updates. Setting up the necessary software is a laborious process that requires a substantial initial investment of effort and ongoing maintenance (e.g. https://training.galaxyproject.org/training-material/topics/admin/).

Over the years, the project has documented and codified parts of this setup, most notably using the Ansible framework, with much of the installation automated. This model of deployment allows a user to assemble various Ansible roles that install parts of the overall stack, configure software interconnections, and target varied infrastructure to meet local requirements. One downside to utilizing Ansible scripts is that, when run at different timepoints, system packages or other dependencies may change, yielding a different deployment.

Here, we describe the Galaxy Helm chart as an alternative deployment option offering bit-for-bit deployment reproducibility and requiring minimal configuration. A Helm chart is a package manager for Kubernetes that simplifies the orchestration of complex applications and services that utilize software containers. Reliance on containers provides bit-for-bit reproducibility regardless of when the system is deployed. This is often an important capability in dynamic environments provisioned on-demand because interoperability between various services is ensured. The Galaxy Helm chart assembles a production grade Galaxy deployment out of pre-built containers, with all necessary services and configurations codified in one place. The chart can be used to install Galaxy on a variety of infrastructure, including a laptop, a workstation, the cloud, and even an HPC system (see link to administrator training above). Internally, the chart automatically sets up the necessary configurations and deploys the needed software, yielding a best-practice Galaxy installation with minimal tuning required by the administrator (see Table 1). Galaxy specific containers are built using existing Ansible roles for Galaxy, while other containers (e.g. database) use community-vetted containers.

**Table 1.** An administrator's overview of key features of the Galaxy Helm chart, highlighting both advantages and disadvantages.

Out-of-the-box features	Description
Precise versioning	Bit-for-bit reproducibility by leveraging software containers
Zero-downtime restarts	Containers rolled over during configuration changes and upgrades with no downtime using Kubernetes features
Automatic failure recovery	Kubernetes liveness and readiness probes ensure that unhealthy containers are automatically restarted
Rapid deployment	∼3 minutes to a running, production-grade Galaxy deployment on an existing Kubernetes cluster
Resumable uploads	Support for resumable data uploads via tusd
Minimal privileges	Jobs run as non-root and only have access to datasets they need; all processes are isolated in containers with RBAC policies
Automatic maintenance	Galaxy maintenance scripts executed on a configurable cron schedule for standard cleanup tasks, including jobs and temporary directories
Interactive tools	Galaxy Interactive Tools will launch and work with no further configuration other than a wildcard DNS mapping
Reference data	Full access to the same Galaxy CVMFS reference data as the usegalaxy.* federation
Full-featured toolset	An extensive and configurable toolset
**Optional features**	
High Availability (HA)	All components can be configured for HA, including web/job handlers, clustered Postgres, and RabbitMQ
Component substitution	Key components substitutable with local or managed versions, e.g. replace built-in Postgres with Amazon RDS
S3 reference data	S3 can be used as an alternative source for reference data, preconfigured to serve as a mirror of CVMFS.
Metrics and monitoring	Metrics can be automatically scraped on a cron schedule into InfluxDB, compatible with (existing) Grafana dashboards
**Disadvantages **	
Familiarity with Kubernetes	Requires administrators to be comfortable with Kubernetes and Helm, which may have a steep learning curve
Less community adoption	Overall, Galaxy community adoption for Ansible is higher, with fewer tutorials and guidance available for the Helm Chart

Despite the seemingly substantial simplification of the complex installation process for Galaxy, the chart is not intended for non-technical users. This is because once deployed, ongoing maintenance and management will require a reasonable understanding of both general system administration as well as Kubernetes administration. To help users get started, tutorials for installation and common administration tasks are available as part of the Galaxy Training Network (Hiltemann *et al.* 2023): https://gxy.io/GTN:T00013 and https://gxy.io/GTN:T00014.

## 2 Galaxy Helm chart

**Figure 1.**
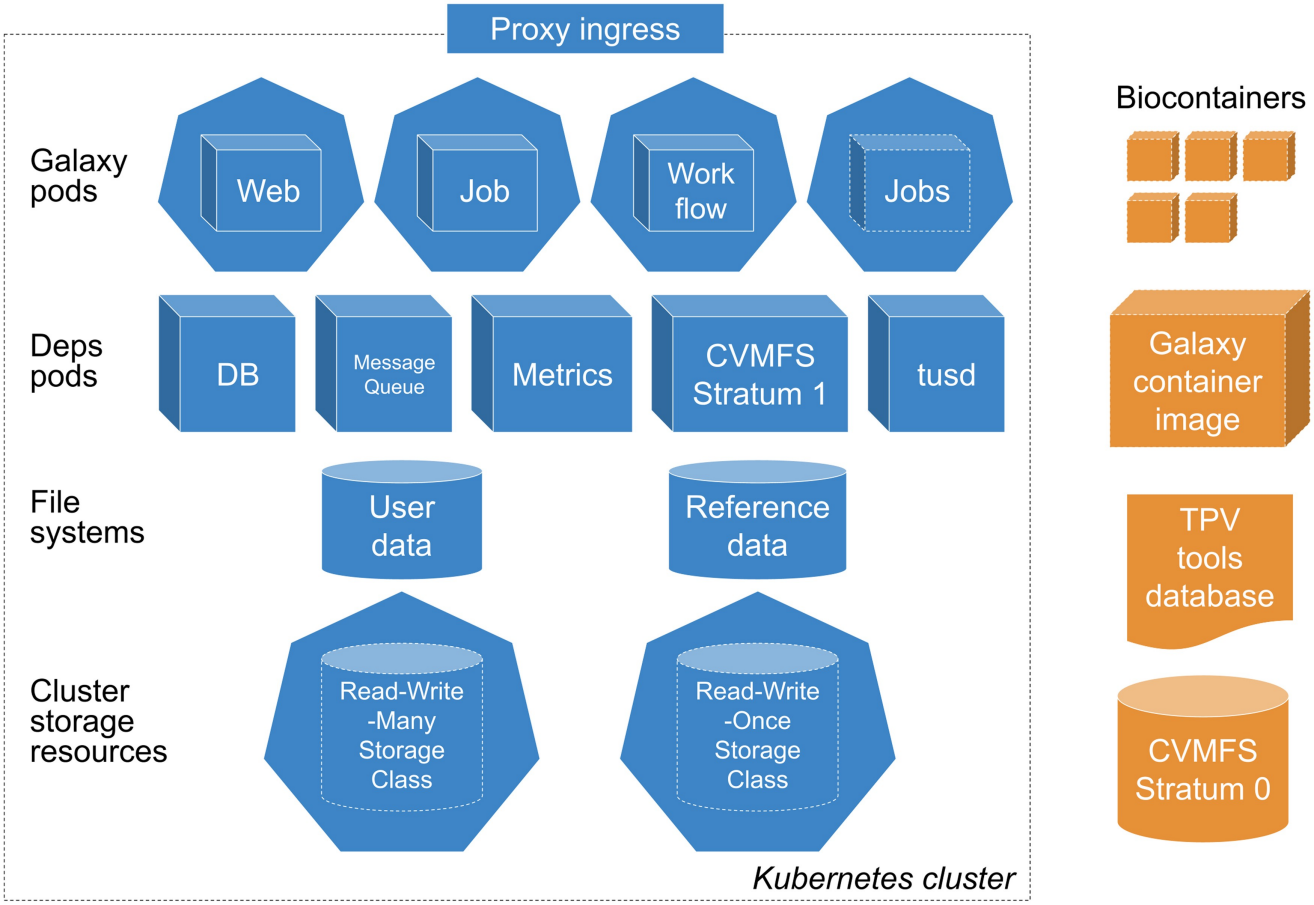
The components of the Galaxy Helm chart, capturing the installation architecture for the Galaxy application. The bottom two layers represent cluster storage resources, visualized as storage classes and corresponding persistent volumes. The next layer-up are dependent applications that are deployed and configured for use by Galaxy. The top layer are the Galaxy application processes, each independently scalable and hot-swappable without downtime. To deliver a comprehensive installation, the chart relies on several community resources. Visualized in the right-most column, these include tool containers from Biocontainers, the Galaxy container image used by Galaxy processes, default resource configuration for tools as captured in the Total Perspective Vortex (TPV) shared database (Goonasekera *et al.*, 2023), and a centrally managed repository of Galaxy’s reference data on CVMFS.

The Galaxy Helm chart utilizes the standard Helm chart structure, capturing (i) chart metadata, (ii) a list of dependencies, (iii) application-specific templates, and (iv) overridable configuration values that interconnects all the components. In this section we describe these in turn, with the overall structure and a recommended architecture for Galaxy installation visualized in Fig. 1. The chart explicitly specifies all software and container versions across the entire deployment stack, yielding a maximally reproducible system.

**Chart metadata:** captures the application name, version, and description.

**Dependent charts:** specify external software dependencies that run alongside Galaxy. These include: a database (PostgreSQL), a message queue (RabbitMQ and Celery), a data upload server (tusd), a reference data client (CVMFS client (Aguado Sanchez *et al.* 2008)), a metrics service (InfluxDB and Grafana), and a web proxy (Nginx). The Galaxy chart specifies all the details for running these dependencies, including the origin of their respective chart, the chart and software version, and any configurations to integrate with Galaxy. Examples of the configurations include communication protocol names and ports, the necessary compute and memory resources that need to be allocated to the service, recommended service timeouts or proxy buffer settings, and file system mount options to name a few. Collectively, this information captures operational knowledge for deploying these services without much experimentation required by the chart user. The chart also supports disabling of dependent services in case the user has a resource available already. For example, instead of the chart automatically deploying a PostgreSQL server, a user can link to an external database service instead.

**Galaxy application processes:** There are three main processes necessary to run the Galaxy application: *web*, *job*, and *workflow*, which are codified as dedicated Kubernetes deployments. The *web handler* is responsible for handling user requests; the *job handler* is responsible for preparing, submitting, and monitoring jobs submitted by users; and the *workflow handler* is responsible for processing workflow submissions before handing off individual jobs to the job handler. Each of the handlers can be independently scaled by adding or removing containers to accommodate workload size and fluctuation. Each handler also implements health checks, including a startup probe (ensuring a process has successfully started) and a continuous liveness probe (ensuring the process operates as expected). Probes are particularly useful when rolling out configuration changes because they facilitate zero downtime upgrades: the system keeps the old process running until a new one is deemed functional so that failed upgrades do not cause downtime. In principle, rollbacks are supported but given the complexity of the Galaxy application and its dependence on external data (e.g., database), rollbacks must be executed with great caution.

**Runtime environment for jobs:** The chart implements the necessary templates to create a runtime environment for jobs submitted by Galaxy users. Specifically, it implements RBAC controls that limit job’s access to the Kubernetes cluster. It also mounts only the relevant data directories into the job container. This contrasts with a traditional Galaxy installation where a job has access to the entire user data folder. The Galaxy ecosystem contains over 10 000 community-contributed tools, making such safeguards against any malicious tools a significant security boon.

**Additional configuration:** The final piece of the deployment is the configurations for all the processes. The chart captures all the necessary operational values, ensuring disks are mounted in relevant containers, ports are opened and set, passwords are defined, etc. The chart also captures a set of recommended values for running the Galaxy application, such as loading the default set of tools and reference data, adding tools that produce graphical outputs to a relevant allow list, managing job cleanup, etc Just about any configuration value can be overridden or new ones added. All the templates for Galaxy processes also support injection of initialization containers, commands, or additional data volumes. This allows the user to specify custom startup operations, such as defining additional environment variables or setting up shared data volumes. Finally, the chart is configured to support Galaxy’s Interactive Tools (Grüning *et al.* 2017) out of the box, which only requires DNS wildcard mapping as a manual administrative step.

## 3 Adoption and use cases

In this section, we describe three different scenarios where the Galaxy Helm chart has been adopted, highlighting its production-grade quality and versatility.

### 3.1 Galaxy on AnVIL

AnVIL is a NHGRI-sponsored data commons where a versatile suite of data analysis applications is made available alongside many popular cohorts of genomic data (Schatz *et al.* 2022). AnVIL operates under FedRAMP-High security certification (Taylor 2014), allowing the platform to host protected datasets. Due to this certification and consequential security implications of software running on the platform, deploying Galaxy on AnVIL required a unique deployment model. Namely, on AnVIL, each user must launch a private instance of Galaxy, and this must be done in minutes within each data workspace users have. This means that even a single user of AnVIL may have multiple, independent instances of Galaxy running. Collectively, this implies there can be hundreds of Galaxy instances running on AnVIL at any point in time, each with its own set of services, infrastructure, and relevant configurations. To support this environment, each Galaxy instance needs to be easy to deploy, robust, and self-managed.

Galaxy on AnVIL has been deployed using the Galaxy Helm chart and has been running in production since April 2021 with hundreds of instances launched by users. The system has been remarkably robust with minimal support interventions, indicating the success of the adopted model.

### 3.2 Training infrastructure for the Bioconductor project

The Bioconductor project develops and distributes some of the most popular software packages for biomedical data analysis in the world (Huber *et al.* 2015). Used in the R programming language, these packages have been downloaded >35 million times from >750 000 unique IPs in 2023 alone. The software is accompanied by a training program, Bioconductor Carpentries, which prepares and delivers educational materials through a worldwide network of trainers. Members of the Bioconductor community can also create and distribute workshops, notably at community conferences. A critical requirement for these workshops is the availability of scalable compute infrastructure. To effectively participate in learning, each workshop participant must have access to their own installation of relevant Bioconductor packages. With many participants coming from low-resourced institutions or regions, finding a computer capable of running the necessary software can become a barrier. Even with a requisite computer is available, differences in operating systems or available storage and memory lead to setup complications, creating delays during workshops or an inability to participate altogether.

In response to these requirements, the Bioconductor project adopted the Galaxy Helm chart as a method for deploying Galaxy and its Interactive Tools environments (Grüning *et al.* 2017), used to deliver the necessary infrastructure for the training participants. This system is available at *workshop.bioconductor.org* and its capacity is scaled up for coordinated community events. In 2023, the project organized 41 hands-on workshops where >1 500 environments were launched by nearly 200 people from dozens of countries. The response was remarkably positive, with praise and excitement from trainers and participants alike requesting to use the platform at future events.

### 3.3 Blueprint for running Galaxy on AWS

Independent installations of Galaxy on commercial cloud providers have been available since 2010 as a method for researchers and labs to easily obtain access to infrastructure and software with no upfront investment (Afgan *et al.* 2011, 2019). Galaxy on AnVIL is an example of such an environment. Recently however, engineers from Amazon Web Services (AWS) implemented an AWS-native blueprint for deploying Galaxy on AWS: https://github.com/aws-solutions-library-samples/guidance-for-galaxy-on-aws. The deployment relies on the Galaxy Helm chart with many dependent services being swapped out for AWS services, such as the database and the message queue. The implementation also adds support for cluster backups and multi-zone replication. While we do not have any insight into the adoption of the offering, the Galaxy Helm chart capabilities appear to be comprehensive enough to be offered in a commercially supported environment. The AWS engineers implemented the capability entirely on their own without requiring any changes to the chart, demonstrating its flexibility.

## References

[btae486-B1] Afgan E , Lonie A, Taylor J et al CloudLaunch: discover and deploy cloud applications. Future Gener Comput Syst 2019;94:802–10. 34366521 10.1016/j.future.2018.04.037 PMC8340934

[btae486-B2] Afgan E , Baker D, Coraor N et al Harnessing cloud computing with galaxy cloud. Nat Biotechnol 2011;29:972–4. 22068528 10.1038/nbt.2028 PMC3868438

[btae486-B3] Aguado Sanchez C , Blomer J, Buncic P et al 2008. CVMFS—a file system for the CernVM virtual appliance. ui.adsabs.harvard.edu.

[btae486-B4] Galaxy Community The galaxy platform for accessible, reproducible, and collaborative data analyses: 2024 update. Nucleic Acids Res 2024;52:W83–W94. 38769056 10.1093/nar/gkae410 PMC11223835

[btae486-B5] Goonasekera N , Bromhead C, Gladman S et al Right-sizing compute resource allocations for bioinformatics tools with Total Perspective Vortex, 2023, arXiv [cs.DC].

[btae486-B6] Grüning BA , Rasche E, Rebolledo-Jaramillo B et al Jupyter and galaxy: easing entry barriers into complex data analyses for biomedical researchers. PLoS Comput Biol 2017;13:e1005425. 28542180 10.1371/journal.pcbi.1005425 PMC5444614

[btae486-B7] Hiltemann S , Rasche H, Gladman S, Galaxy Training Network et al Galaxy training: a powerful framework for teaching!. PLoS Comput. Biol 2023;19:e1010752. 36622853 10.1371/journal.pcbi.1010752 PMC9829167

[btae486-B8] Huber W , Carey VJ, Gentleman R et al Orchestrating high-throughput genomic analysis with bioconductor. Nat Methods 2015;12:115–21. 25633503 10.1038/nmeth.3252 PMC4509590

[btae486-B9] Moreno P , Pireddu L, Roger P et al 2018. Galaxy-Kubernetes integration: scaling bioinformatics workflows in the cloud.

[btae486-B10] Schatz MC , Philippakis AA, Afgan E et al Inverting the model of genomics data sharing with the NHGRI genomic data science analysis, visualization, and informatics lab-space. Cell Genom 2022;2. 10.1016/j.xgen.2021.100085 PMC8863334 35199087

[btae486-B11] Taylor L. FedRAMP: history and future direction. IEEE Cloud Comput 2014;1:10–4.

